# The “Hand as Foot” teaching method for ocular anatomy: an embodied learning approach

**DOI:** 10.3389/fmed.2026.1883947

**Published:** 2026-09-03

**Authors:** Muge Qi, Xianrong Yang

**Affiliations:** The Affiliated Hospital of Inner Mongolia Medical University, Hohhot, China

**Keywords:** education, embodied cognition, medical, ophthalmic education, teaching method

## Abstract

**Background:**

Ocular anatomy is difficult to teach due to complex three-dimensional relationships. Traditional two-dimensional methods limit comprehension, while virtual simulation is often unavailable in underdeveloped regions. The “Hand as Foot” teaching method uses hand gestures to simulate anatomical structures and has been effective in other disciplines. This study introduces this method into ocular anatomy teaching to address three core obstacles: layered wall structure, dynamic circulation/lens accommodation, and retinal functional mapping.

**Methods:**

We introduced the “Hand as Foot” teaching method into an ocular anatomy course at a medical university in northern China. A total of 91 students participated: 45 in the control group and 46 in the intervention group. The intervention group learned key features (eyeball wall layers, aqueous humor circulation, retinal imaging) using this method. Learning outcomes were assessed by theoretical knowledge test, clinical skills assessment, and satisfaction questionnaire. Results No significant baseline differences existed between groups. The intervention group scored significantly higher than the control group on the theoretical knowledge test (90.04 ± 5.00 vs. 85.84 ± 6.13; *p* = 0.001) and the clinical skills assessment (87.28 ± 3.16 vs. 84.93 ± 4.03; *p* = 0.003). Overall teaching satisfaction was 91.3% in the intervention group vs. 73.33% in the control group (*p* = 0.024).

**Conclusion:**

The “Hand as Foot” teaching method is easy to implement, and achieved higher scores in both theoretical understanding and clinical skills in ocular anatomy. It provides a practical, engaging alternative for ophthalmic anatomy education, particularly in resource-limited settings.

## 1 Innovation in medical education

The educational objectives of the ophthalmology curriculum are threefold: (a) to familiarize students with the basic theories of visual system disorders, including their onset, progression, and prognosis, treatment, prevention, and rehabilitation; (b) to equip students with theoretical knowledge and clinical skills related to ocular anatomy and common ophthalmic diseases; and (c) to enable students to apply this knowledge in clinical practice. Presently, two major challenges are confronting ophthalmology education. First, the curriculum is extensive, but class time is limited. Despite the intricacy of the eye’s anatomy and the wide spectrum of ophthalmic diseases, instructors face challenges in providing comprehensive coverage of all subjects within the allocated time. Consequently, instruction often emphasizes only core concepts, which prevents students from independently constructing a systematic clinical reasoning framework. Secondly, the curriculum places significant emphasis on anatomical and physiological mechanisms, thereby imposing substantial demands on students’ spatial visualization and logical reasoning skills. Consequently, students often perceive ophthalmology content as “invisible, incomprehensible, and difficult to retain.”

In recent years, multimedia has been incorporated into teaching methods, and the use of big data and social media has led to further improvements in learning outcomes. Nonetheless, conventional methods that depend on static images and models are constrained in their capacity. These methods offer solely two-dimensional visual aids and are deficient in engagement and experiential learning opportunities. Despite the integration of models such as BOPPPS and Problem-Based Learning (PBL) in ophthalmology education, the reliance on passive information dissemination has resulted in an “disconnected” learning experience. Consequently, contemporary methodologies are inadequate in addressing the need for more interactive, student-centered pedagogical approaches. Virtual reality (VR), a key form of virtual simulation, is a learning tool that creates interactive models and clinical simulations. By enabling users to interact with people or virtual objects (1), VR facilitates human-environment interaction, stimulates perception, and provides an immersive experience, thereby improving undergraduate medical education (2). Embodied cognition has emerged as a major research focus in cognitive science. This theory challenges mind-body dualism positing that cognition arises from body-environment interaction, a concept that closely aligns with the principles of virtual simulation technology. However, the adoption of virtual reality (VR) technology remains constrained in underdeveloped regions of western China. In this context, the “Hand as Foot” teaching method pioneered by an orthopedic teaching team at a university-affiliated hospital offers a practical, low-cost alternative. Previous research on ophthalmology coursework and clinical training (3–5) have shown that students often lack a dynamic understanding of ocular anatomy due to its three-dimensional complexity. Moreover, ocular anatomy receives little attention during clinical rotations (6). Embodied cognition emphasizes mind-body interaction in cognitive processes, providing a theoretical basis for designing concrete, learner-centered teaching methods. We systematically adapted the orthopedic-derived “Hand as Foot” method to ocular anatomy, developing an embodied teaching framework informed by this theory. This approach is designed to enhance the interactivity of ocular anatomy instruction and facilitate student mastery of relevant knowledge.

## 2 The origins of the “Hand as Foot” teaching method

The “Hand as Foot” teaching method, developed by an orthopedic teaching team, uses hand gestures to transform abstract anatomical concepts into three-dimensional representations, embodying the principle that “the body participates in cognitive construction.” Students simulate ocular structures with their own upper limbs to develop spatial understanding. By comparing upper limb and ocular anatomy, students enhance their interest, retention, and comprehension. Interaction has been shown to reduce instructional time and improve understanding of textbook concepts. Combining illustrations with PowerPoint presentations further enhances clinical teaching.

### 2.1 Participants

This study was conducted at a medical university in northern China between September 2025 and January 2026. A total of 91 second-year clinical medicine students enrolled in the compulsory ophthalmology course participated. This was a non-randomized comparison of two intact class cohorts. Class A (*n* = 45) served as the control group and received traditional lecture-based instruction. Class B (*n* = 46) served as the intervention group and received the “Hand as Foot” teaching method. Both cohorts were taught by the same instructor, received the same total contact hours (6 h), and covered the same curriculum content.

### 2.2 Study design

The control group received conventional lecture-based instruction using PowerPoint presentations, static diagrams, and anatomical models. For the intervention cohort, we integrated the “Hand as Foot” teaching method with PPT slides and anatomical models as a complementary approach for the ocular anatomy course. Given the course’s emphasis on three-dimensional spatial relationships, we used hand gestures to simulate the key anatomical structures and their functions, covering three core learning obstacles: static structure, dynamic process, and functional mapping. Taking the teaching of glaucoma (Chapter 10 of Ophthalmology) as an example, we further applied the same hand-gesture simulations to illustrate the impaired aqueous humor outflow pathway and the resulting intraocular pressure elevation, thereby reinforcing students’ understanding of both normal anatomy and its pathological changes. The intervention was delivered in three 2-h sessions over 1 week. The acute angle-closure glaucoma (AACG) sequence was integrated into the final session. It followed the separate teaching of the three core obstacles and consisted of instructor demonstration (10 min), student hands-on practice (15 min), and group debriefing with feedback (5 min). As part of the complete educational intervention, the reported outcomes reflect overall effectiveness and should not be interpreted as evidence of the independent effect of the AACG sequence alone.

### 2.3 Data analysis

The primary outcome was the clinical skills assessment score; secondary outcomes were theoretical knowledge and student satisfaction. The theoretical knowledge test and clinical skills assessment were developed from the teaching syllabus and institutional item bank, with questions selected by the research team. The theoretical knowledge test comprised 40 multiple-choice questions covering ocular anatomy, aqueous humor physiology, lens and accommodation, retinal structure and function, and clinical applications (10, 8, 8, 6, and 8 items, respectively; total 100 points). The clinical skills assessment used a standardized checklist (see Supplementary material; total 100 points). Both assessments were locally developed by the teaching team. Two trained assessors, blinded to group allocation, scored the assessments. Satisfaction was assessed using a three-point scale (very satisfied, satisfied, dissatisfied), developed by the research team. Normality was assessed using the Shapiro-Wilk test and homogeneity of variance using Levene’s test. Between-group comparisons were performed using independent-samples *t*-tests. No adjustment for multiple comparisons was made, as the outcomes were pre-specified and limited in number.

## 3 The “Hand as Foot” teaching method in the systematic instruction of eye anatomy

For clinical undergraduate medical students at a university in northern China, we integrated the “Hand as Foot” teaching method with PPT slides and anatomical models as a complementary approach for the ocular anatomy course. Given the course’s emphasis on three-dimensional spatial relationships, we used hand gestures to simulate the key anatomical structures and their functions, covering three core learning obstacles: static structure (the layered wall), dynamic process (aqueous humor circulation and lens accommodation), and functional mapping (retinal imaging). Taking the teaching of glaucoma (Chapter 10 of Ophthalmology) as an example, we further applied the same hand-gesture simulations to illustrate the impaired aqueous humor outflow pathway and the resulting intraocular pressure elevation, thereby reinforcing students’ understanding of both normal anatomy and its pathological changes.

### 3.1 Addressing the first obstacle: the layered eyeball wall

Understanding the anatomy and physiology of the visual organs, as well as the diagnosis and treatment of common ophthalmic diseases, is essential for clinical practice. The eyeball wall comprises three concentric tunics: the outer fibrous tunic, the middle vascular tunic, and the inner nervous tunic (7–9). The baseline gesture-to-anatomy mappings for the static anatomical structures described in sections “3.1 Addressing the first obstacle: the layered eyeball wall”–“3.4 Addressing the first obstacle: the layered eyeball wall” are summarized in Table 1.

**TABLE 1:** Summarizes the baseline gesture-to-anatomy mapping used in the following sections.

Gesture	Anatomical structure
Both hands in cradling position, tilted slightly to the left	Eyeball wall (three tunics)
Tip of the middle finger	Cornea
Dorsum of the hand	Sclera
Forearms	Optic nerve
Space between the forearms	Supplying blood vessels
Index finger	Iris
Central palm area	Choroid
Base of the thumb	Ciliary body
Inner surface of the palms	Retina
Space between the middle and index fingers	Anterior chamber
Tip of the index finger	Lens
Space between the two hands	Vitreous humor
Left palm base (central depression)	Fovea centralis
Right hand bent	Optic disk

**FIGURE 1:**
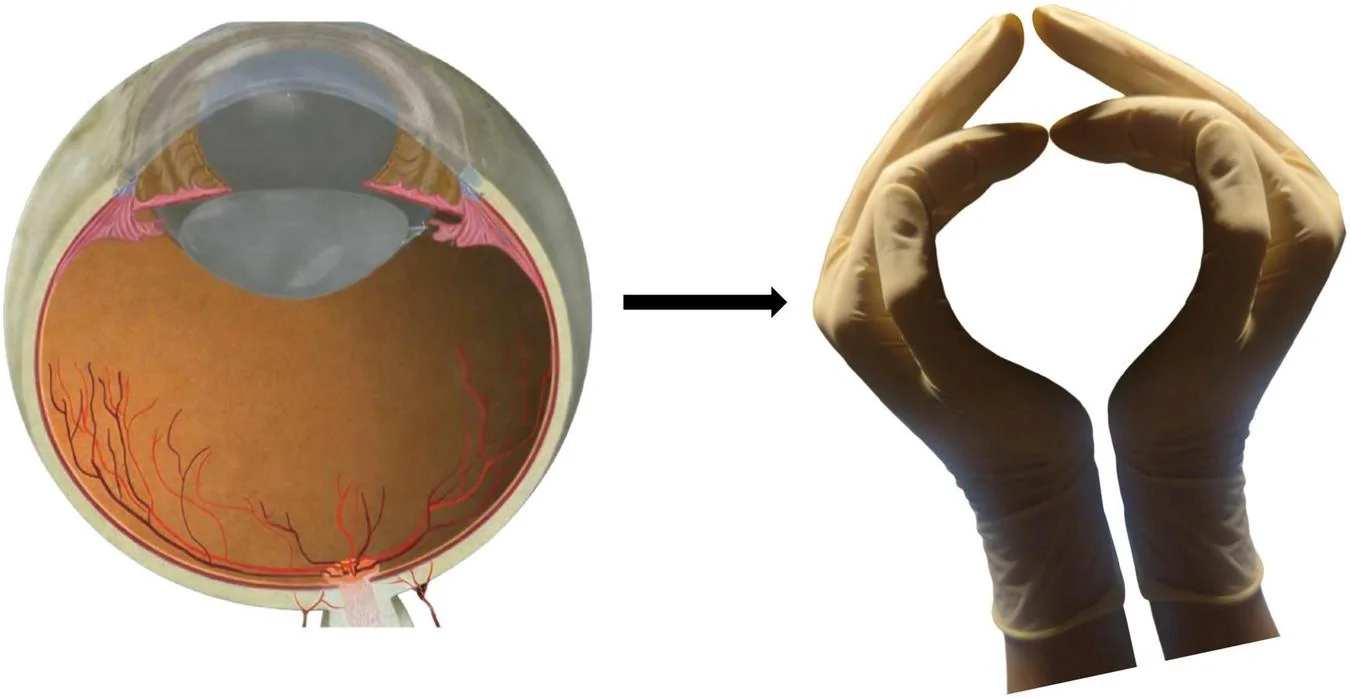
Model building. The cradling hand posture simulates the three-layered eyeball wall. (The hand images are of the authors. All illustrations were created by the authors). Adapted with permission from Ophthalmology by Yang PZ, Fan XQ, editors. Source: Yang PZ, Fan XQ, editors. Ophthalmology. 9th ed. Beijing: People’s Medical Publishing House; 2018, Chapter 2, Figure 2-1.

**FIGURE 2:**
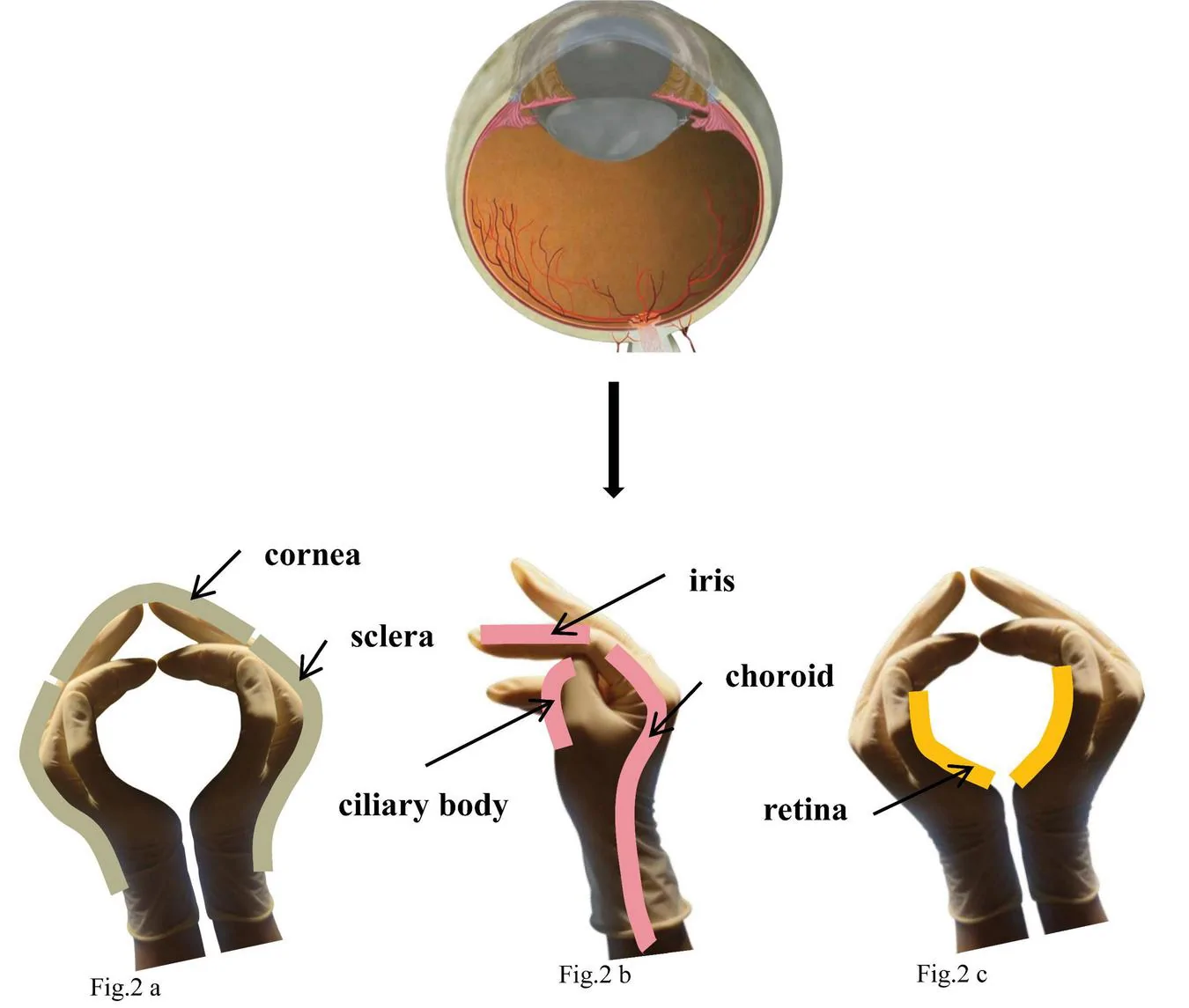
Simulation of the eyeball wall. **(a)** Outer tunic; **(b)** uveal tract; **(c)** retina. Adapted with permission from Ophthalmology by Yang PZ, Fan XQ, editors. Source: Yang PZ, Fan XQ, editors. Ophthalmology. 9th ed. Beijing: People’s Medical Publishing House; 2018, Chapter 2, Figure 2-1.

To simulate the eyeball’s anatomy, we hold both hands in a cradling position tilted slightly to the left (Figure 1). The outer surface of each hand’s back represents the lateral wall (Figure 2a). The tip of the middle finger is the cornea, the rest of the back is the sclera, the forearms are the optic nerve, and the space between the arms represents the supplying blood vessels. The inner surface of the palms represents the retina (Figure 2c), and the center of the palms represents the uvea (vascular pigment layer), a concept that is often difficult to teach (10). The uvea consists of three continuous parts from posterior to anterior: the choroid, ciliary body, and iris. We use an additional gesture to detail the uvea (Figure 2b), the index finger is the iris, the central palm below the index finger is the choroid, and the base of the thumb is the ciliary body. This innovative visualization method is the first step toward a deep understanding and effective teaching of the subject.

### 3.2 Physiological anatomy of the intraocular contents

**FIGURE 3:**
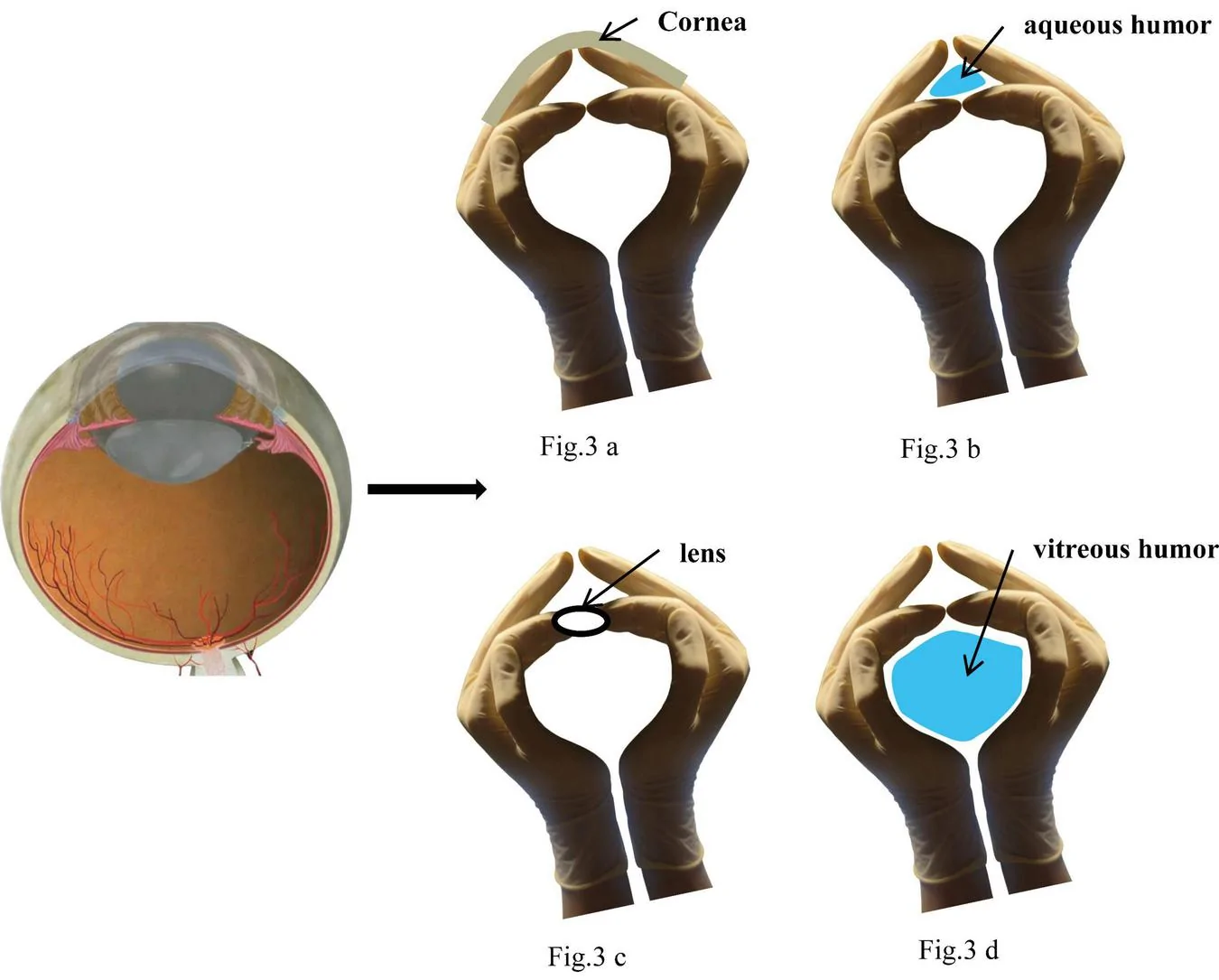
Demonstration of the intraocular components. **(a)** cornea; **(b)** anterior chamber; **(c)** lens; **(d)** vitreous body. Adapted with permission from Ophthalmology by Yang PZ, Fan XQ, editors. Source: Yang PZ, Fan XQ, editors. Ophthalmology. 9th ed. Beijing: People’s Medical Publishing House; 2018, Chapter 2, Figure 2-1.

The eye is a vital sensory organ that plays a central role in how individuals perceive and interact with their environment. Optometrists and ophthalmologists collaborate to manage refractive errors and play a pivotal role in preventing and controlling myopia in children and adolescents. Optometry knowledge constitutes a fundamental component of ophthalmology, providing the theoretical underpinnings and practical competencies indispensable for the professional mastery of both disciplines. The contents of the eyeball consist of three transparent substances: aqueous humor, the lens, and the vitreous humor. These substances form the pathway for light to reach the retina; together with the cornea, they constitute the eye’s refractive media. We use hand gestures to sequentially demonstrate the structural characteristics and functional relationships of these refractive media. As shown in Figure 3a, the tip of the middle finger simulates the cornea, the first window through which light enters the eye. As the primary refractive medium, the cornea’s curvature and transparency directly affect image quality. The space between the middle and index fingers represents the anterior chamber, which is filled with aqueous humor (Figure 3b). The dynamic circulation of aqueous humor provides nutritional support and facilitates metabolic processes for the avascular cornea and lens. The blood-aqueous barrier is a physiological mechanism that ensures the transparency of the refractive media within the eye by preventing the presence of blood cells and minimal levels of protein in the aqueous humor.

The tip of the index finger simulates the lens (Figure 3c). The lens capsule plays a key role in metabolic transport. In the event of capsule damage or alterations to aqueous humor composition, lens proteins undergo denaturation, leading to lens opacification. This process ultimately results in cataract development. The suspensory ligaments of the lens originate from the ciliary body and attach to the anterior and posterior capsules surrounding the equator of the lens. The ciliary body is represented by both hands, with the fingers flexed and extended to simulate the tension and relaxation of the suspensory ligaments. When observing the distance, the hands are in a flexed position, indicating that the ciliary muscles are relaxed and the suspensory ligaments are taut. This results in the lens being pulled into a flattened shape. When the gaze shifts to a nearby object, the hands extend, indicating that the ciliary muscles contract and the suspensory ligaments relax. This allows the lens to increase its anterior curvature through its own elasticity, thereby enhancing refractive power. This dynamic process occurs concurrently with the constriction of the pupil, ensuring that light is focused on the most sensitive area of the retina.

The space between the hands simulates the vitreous humor (Figure 3d), a gelatinous substance with slow metabolism and a non-regenerative nature. As individuals age, the collagen fiber scaffold of the vitreous humor undergoes a process of collapse or contraction, which can potentially result in vitreous liquefaction and posterior detachment. This clinical condition is characterized by the presence of floaters.

By transforming abstract refractive media into intuitive spatial models through hands-on simulation, students can develop an understanding of the anatomical location, physiological function, and clinical relevance (e.g., cataracts, vitreous liquefaction, refractive errors) of each refractive medium. In summary, the complexity of the eye’s anatomy poses a significant challenge for medical students attempting to memorize every detail. Minor eye injuries can induce structural abnormalities, leading to visual impairment or loss. This phenomenon impacts daily life, academic pursuits, and professional activities, resulting in substantial losses for individuals, families, and society. The “Hand as Foot” teaching method facilitates retention of ocular knowledge, provides an intuitive understanding of abstract anatomical concepts, and enables clinical application (11).

### 3.3 Addressing the second obstacle: dynamic processes

**FIGURE 4:**
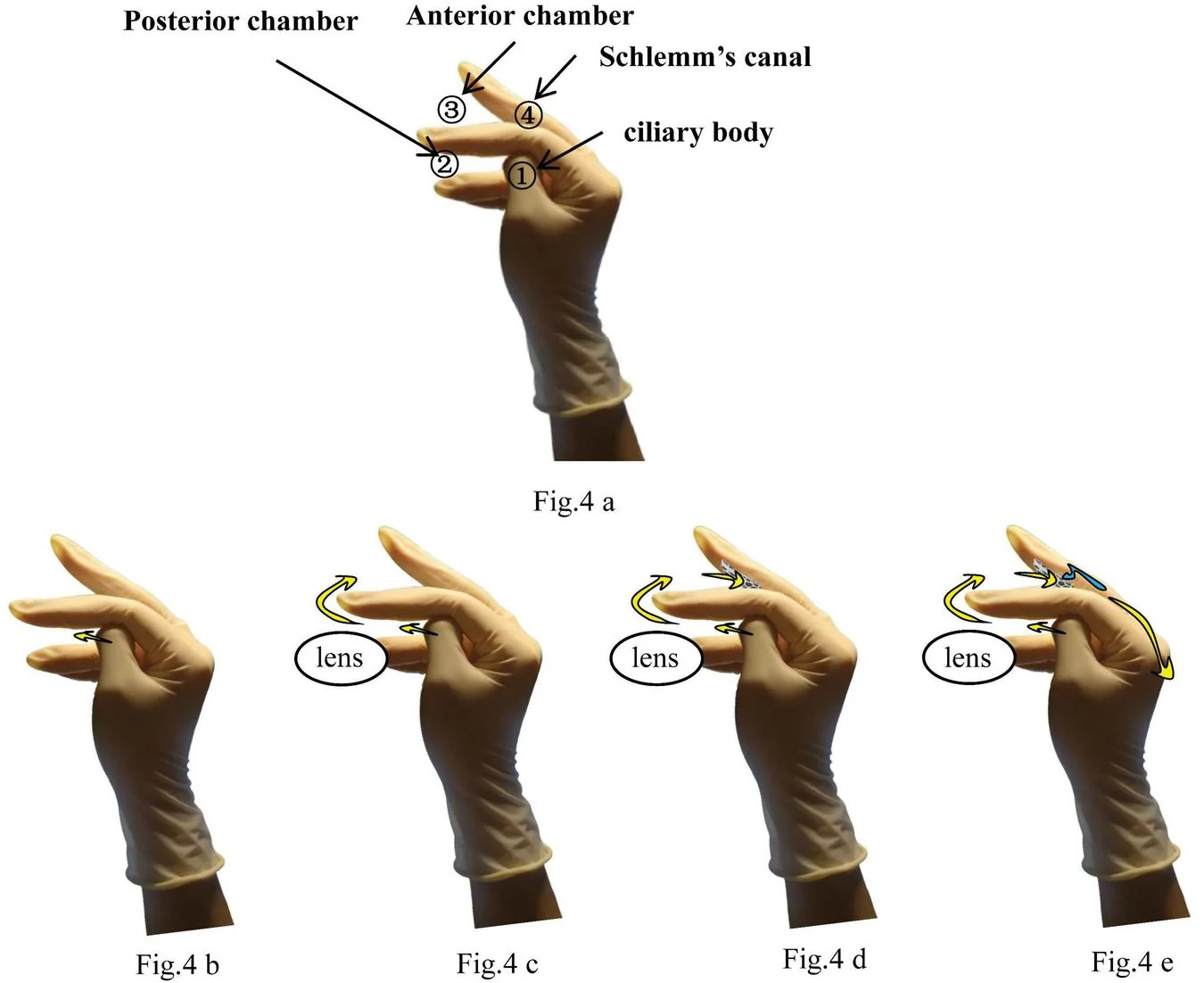
Simulation of aqueous humor dynamics. **(a)** Ciliary body → posterior chamber; **(b)** Pupil → anterior chamber; **(c)** Trabecular meshwork → Schlemm’s canal; **(d)** Episcleral veins → **(e)** systemic circulation.

The limbus is anatomically important as the location of the anterior chamber angle and aqueous drainage system. It is also a key clinical landmark for intraocular incisions and histologically, it contains corneal stem cells. Together, these properties give the limbus its educational and clinical value (12). The anterior chamber angle lies at the junction of the peripheral cornea and the iris root, serving as the main outlet for aqueous humor drainage. Produced by the ciliary epithelium, aqueous humor supplies nutrients, clears metabolic waste, and helps regulate intraocular pressure to maintain the eyeball’s structural integrity. Poor visual habits may raise intraocular pressure, resulting in chronic glaucoma or acute angle-closure glaucoma, with headache and blurred vision as common symptom. We further elaborate on this using Figure 2b. The detailed steps are shown in Figure 4a (steps ➀–➃). Within the anterior chamber angle, the following structures are arranged in order from anterolateral to posteromedial: the Schwalbe line, the trabecular meshwork, the scleral spur, the ciliary crests, and the root of the iris. Together, these structures form the outflow pathway for aqueous humor. Aqueous humor is produced by the ciliary body and enters the posterior chamber (Figure 4b). It then flows through the pupil into the anterior chamber (Figure 4c), passes from the trabecular meshwork into Schlemm’s canal (Figure 4d), and finally drains through the collecting ducts and aqueous veins into the anterior ciliary veins on the scleral surface, returning to the systemic circulation (Figure 4e).

Aqueous humor drains primarily through the conventional trabecular pathway, with a fraction exiting via the unconventional uveoscleral pathway (13). Through this demonstration, students can better understand the pathophysiological mechanisms related to aqueous humor.

### 3.4 Addressing the third obstacle: functional mapping of the retina

In past teaching, we often used camera structure and imaging principles to explain retinal imaging by analogy. However, the retina is not a uniform photosensitive layer. The structural differences across its various regions determine their corresponding functional characteristics and clinical significance. The fovea centralis contains only cone cells, which confer acute visual acuity. When the macula is diseased, visual acuity declines significantly. Beyond the fovea, the density of cone cells decreases rapidly. At a distance of approximately 5 mm from the fovea, the density of rod cells reaches its peak, gradually decreasing toward the periphery. Rod cells are responsible for low-light vision. Therefore, when the peripheral retina is affected by disease, damage to these cells can lead to night blindness.

**FIGURE 5:**
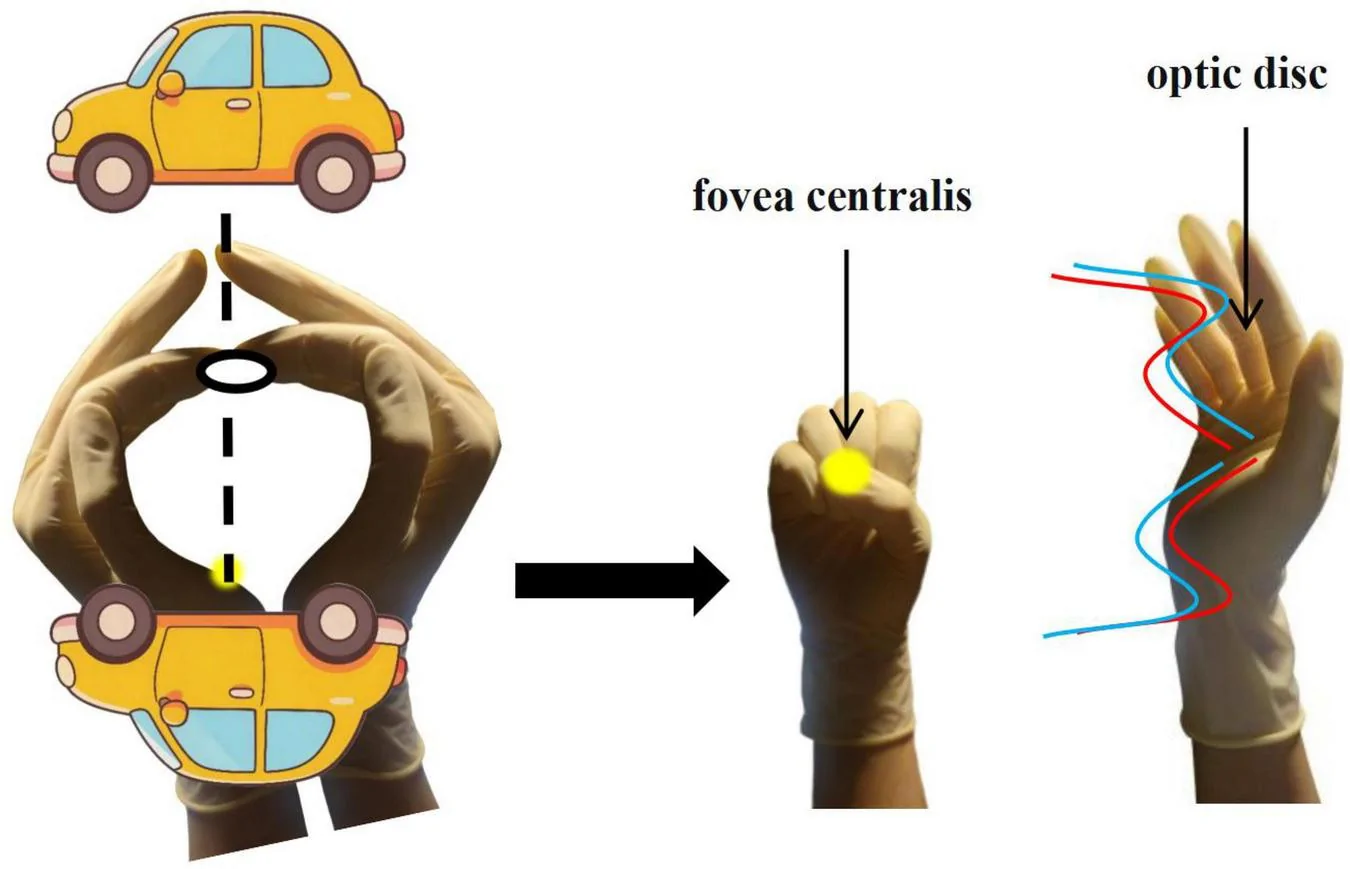
Simulation of fundus imaging. (Left hand, fovea centralis; bent right hand, optic disk).

In this section, we build upon Figure 3c to make retinal imaging more intuitive. Specifically, the left hand simulates the fovea, while the right hand is bent to represent the optic disk (Figure 5), transforming the abstract retinal structure into a tangible spatial model. Localization of the macular region is a critical component of clinical ophthalmic imaging. A comprehensive study of its morphological characteristics helps explore the mechanisms of eye disease onset and progression, predicts early pathological changes, and enables precise staging (14). Furthermore, accurate localization of the optic disk and fovea supports clinical decision-making (15). Unlike the photoreceptive regions, the optic disk is the origin of the optic nerve, formed by the convergence of nerve fibers. Since there are no photoreceptor cells at this site, it lacks visual function and creates the physiological blind spot in the visual field. Using the “Hand as Foot” teaching method, students gain a spatial understanding of key fundus structures through hands-on practice. This lays a solid foundation for later interpreting fundus images and understanding the pathological mechanisms of macular diseases and optic disk lesions.

The above subsections addressed each obstacle independently. However, clinical reasoning often requires simultaneous integration of all three. The following subsection presents a sequential hand gesture that unifies the three obstacles using acute angle-closure glaucoma (AACG) as a systemic disease model.

### 3.5 Integrating the three obstacles into a systemic clinical reasoning pathway: the acute angle-closure glaucoma model

This section uses a separate dynamic gesture system to simulate pathological progression. Unlike the static mapping for normal anatomy (Table 1), it re-assigns hand regions to represent disease-related changes (Table 2). This four-step sequence was delivered to the intervention group as an integrated clinical reasoning exercise, unifying the three previously taught obstacles into a single systemic pathway. Acute angle-closure glaucoma (AACG) was selected as the model disease to link anatomy, pathophysiology, and clinical consequences.

**TABLE 2:** Dynamic gesture adaptations in the acute angle-closure glaucoma (AACG) clinical reasoning sequence.

Step	Gesture	Simulated pathology
Step 1	Cradling position, fingers slightly apart	Normal anatomy: open angle, normal aqueous circulation
Step 2	Middle and index fingertips brought together, wrists slightly flexed	Narrow angle, increased outflow resistance, physiological pupillary block tendency
Step 3	Fingertips close; middle finger pushed forward and bulged	Pupillary block, iris bombé, angle closure, IOP spike
Step 4	Palms face upward; thumb presses into opposite palm center	Sustained IOP elevation compressing optic nerve head → optic nerve damage, visual field defect

Acute angle-closure glaucoma was selected because its pathophysiology naturally links a narrow anterior chamber angle (obstacle 1), arrested aqueous outflow due to pupillary block (obstacle 2), and optic nerve damage from sustained elevated intraocular pressure (obstacle 3). The disease progression can be simulated through continuous hand posture changes, each corresponding to a specific pathological stage. The gesture sequence builds upon the basic cradling hand position described in section “3.1 Addressing the first obstacle: the layered eyeball wall” (Figure 1) and follows the finger assignments already established in previous subsections.

#### 3.5.1 Step 1 -normal anatomy and aqueous circulation

Both hands are held in the cradling position with fingers slightly apart (Figure 1), dorsal surfaces outward and palmar surfaces inward. This posture represents the normal three-layered eyeball wall, an open anterior chamber angle, and normal aqueous circulation. This establishes the baseline of normal anatomy and physiology.

#### 3.5.2 Step 2 -narrow angle and increased outflow resistance

Maintaining the cradling posture, the tips of the middle and index fingers are brought together to narrow the interdigital cleft, while both wrists are slightly flexed. This gesture simulates a narrow angle, increased lens-iris contact, and elevated resistance to aqueous outflow (physiological pupillary block tendency). Students are required to identify anatomical risk factors (hyperopia, thickened lens, shallow anterior chamber).

#### 3.5.3 Step 3 -pupillary block and angle closure

Fingertip pressure is increased to completely close the cleft; the middle finger is pushed forward and its middle part is bulged, while the wrists remain flexed. This gesture comprehensively simulates pupillary block, iris bombé, angle closure, complete arrest of aqueous outflow, and a sharp rise in intraocular pressure. Students are required to explain the mechanism of acute AACG attack and understand why mydriatics are contraindicated.

#### 3.5.4 Step 4 -optic nerve damage and visual field defect

Maintaining the closed-angle posture, both hands are rotated so that the palms face upward. The thumb of one hand is pressed firmly into the center of the opposite palm (optic disk area, Figure 5), followed by lightly touching the central depression of the palm (fovea) with the index finger. This action represents high intraocular pressure compressing the optic nerve head, leading to axoplasmic flow arrest, retinal ganglion cell apoptosis, optic disk cupping, and irreversible visual field defect. Students are required to reconstruct the complete causal chain: narrow angle → arrested aqueous outflow → elevated intraocular pressure → optic nerve damage → visual field defect, and to understand the importance of timely intervention (within hours).

After completing the four-step sequence, students are asked to verbally reconstruct the above causal chain. This embodied sequence transforms abstract pathophysiological concepts into a tangible, repeatable bodily experience, thereby integrating the three isolated teaching obstacles into a single systemic clinical reasoning pathway. This directly addresses the core problem raised in the introduction: that students struggle to independently construct a systematic clinical reasoning framework.

## 4 Results

A total of 91 students were enrolled, with 45 in the control group and 46 in the intervention group. All participants completed the study. Baseline demographic characteristics (age and sex) were comparable between the two groups (Table 3). No pre-intervention assessment of prior ophthalmological knowledge, academic ability, motivation, or spatial reasoning was conducted.

**TABLE 3:** Comparisons of general information between two groups.

Group	*n*	Sex (*n*)		Age (year, mean ± SD)
		Male	Female	
Control group	45	29	16	22.89 ± 1.15
Intervention group	46	28	18	22.83 ± 1.18
t/chi-square	–	0.124		0.257
*P*-value	–	0.724		0.798

The intervention group scored significantly higher than the control group on the theoretical knowledge test (90.04 ± 5.00 vs. 85.84 ± 6.13; mean difference: 4.20, 95% CI: 1.86 to 6.54; *p* = 0.001; Cohen’s *d* = 0.75). The intervention group also scored significantly higher on the clinical skills assessment (87.28 ± 3.16 vs. 84.93 ± 4.03; mean difference: 2.35, 95% CI: 0.83 to 3.87; *p* = 0.003; Cohen’s *d* = 0.65) (Table 4).

**TABLE 4:** Comparisons of theoretical and skill assessment between two groups (mean ± SD).

Group	*n*	Theoretical assessment	Skill assessment
Control group	45	85.84 ± 6.13	84.93 ± 4.03
Intervention group	46	90.04 ± 5.00	87.28 ± 3.16
95% CI	–	(1.86, 6.54)	(0.83, 3.87)
Cohen’s d	–	0.75	0.65
*T*-value	–	3.57	3.091
*P*-value	–	0.001	0.003

Overall teaching satisfaction was higher in the intervention group (91.3%) than in the control group (73.33%; χ^2^ = 5.07, *p* = 0.024) (Table 5). Satisfaction was analyzed using a chi-square test after collapsing the three original categories into a binary measure (satisfied vs. dissatisfied).

**TABLE 5:** Comparisons of teaching satisfaction between two group.

Group	Satisfied	%	Dissatisfied	%
	*n*		*n*	
Control group	33	73.33	12	26.67
Intervention group	42	91.3	4	8.7
χ^2^	–	–	–	5.07
*P*-value	–	–	–	0.024

“Satisfied” includes respondents who selected “very satisfied” or “satisfied” on the original three-point scale; “dissatisfied” includes respondents who selected “dissatisfied.”

## 5 Discussion

This exploratory study assessed three primary outcomes: theoretical knowledge, clinical skills, and student satisfaction. The intervention group demonstrated higher scores across all three measures compared with the control group.

The theoretical knowledge test showed a moderate-to-large effect (Cohen’s *d* = 0.75), and the clinical skills assessment showed a large effect (Cohen’s *d* = 0.65). Student satisfaction was also higher in the intervention group (91.3% vs. 73.3%). These findings are consistent with previous studies on gesture-based teaching in anatomy education, which have reported similar effect sizes for knowledge acquisition and skill development (16, 17), and align with earlier reports that experiential and participatory approaches are associated with higher satisfaction ratings (18).

Several mechanisms may explain these observed differences. First, gesture-based simulations engage motor representations that support spatial understanding beyond visual observation (16, 17). When students use their hands to model ocular structures, for example by forming a cradling posture for the eyeball or using finger positions to represent the lens and ciliary body, they are not merely memorizing labels but physically enacting spatial relationships. This sensorimotor engagement has been shown to facilitate mental model construction and improve recall of three-dimensional anatomical configurations (17). Second, physical enactment of dynamic physiological processes, such as aqueous humor circulation or lens accommodation, may strengthen memory formation through multimodal sensory integration that combines visual, tactile, and proprioceptive input (17). Third, embodied analogies may reduce cognitive load by anchoring abstract pathophysiological concepts in familiar physical experiences (19). For example, comparing aqueous humor outflow to a plumbing system may help students connect anatomical structures to their functional consequences without requiring them to hold multiple abstract relationships in working memory simultaneously. Fourth, the sequential gesture described in section “3.5 Integrating the three obstacles into a systemic clinical reasoning pathway: the acute angle-closure glaucoma model” may help learners integrate isolated anatomical facts into a coherent clinical reasoning pathway (19). By physically transitioning through the stages of acute angle-closure glaucoma from normal anatomy to narrow angle, pupillary block, and optic nerve damage, students may embody the causal chain of the disease, which may support retention and application of the underlying pathophysiological concepts. These findings are consistent with previous studies on gesture-based teaching in anatomy education (16, 17), and align with earlier reports that experiential and participatory approaches are associated with higher satisfaction ratings (18).

However, several limitations should be considered. First, this was an exploratory study with a relatively small sample from a single institution, which limits generalizability. The study used a quasi-experimental design with two existing class cohorts; pre-existing differences between cohorts cannot be entirely excluded and may have influenced the observed outcomes. Because the intervention was allocated at the class level with only one class per condition, the intervention effect cannot be statistically separated from class-level confounders. Second, no pre-intervention knowledge assessment was conducted, so baseline comparability in ophthalmic knowledge, academic ability, or spatial reasoning cannot be established. Age and sex comparability does not ensure equivalence in these domains, limiting causal attribution. Third, formal reliability testing was not performed for the assessment instruments, and the satisfaction measure was a simple non-validated questionnaire. Fourth, several potential sources of bias should be noted: the visibly different teaching formats may have introduced performance bias; instructor allegiance and student expectancy effects cannot be excluded; and novelty effects may have particularly influenced satisfaction ratings. These factors, together with the class-level design and absence of baseline assessment, warrant caution in interpretation. Fifth, no long-term follow-up data were collected, so the durability of the observed improvements remains unknown. Future research with larger, multi-center, longitudinal designs and validated instruments is needed to confirm these preliminary findings. Given these limitations, the present findings should be interpreted as exploratory and require confirmation in future investigations.

## 6 Conclusion

The “Hand as Foot” teaching method achieved higher scores theoretical knowledge and clinical skills compared with traditional instruction, with moderate-to-large effect sizes, and was associated with higher student satisfaction. It fosters teacher-student interaction and mutual learning, and offers a practical approach to addressing key teaching challenges in ocular anatomy. Its analogical nature encourages creative exploration, and when used alongside multimedia resources, it supports active learning and knowledge internalization (20, 21).

## Data Availability

The raw data supporting the conclusions of this article will be made available by the authors, without undue reservation.
