# Supplementary material for: The “Hand as Foot” teaching method for ocular anatomy: an embodied learning approach
Source: Front Med (Lausanne). 2026 Sep 3;13:1883947. doi: 10.3389/fmed.2026.1883947 (PMC13581742; doi:10.3389/fmed.2026.1883947)
Supplement: Supplementary file 1 [file Table_1.docx]

- **Supplementary File – Clinical Skills Assessment Checklist**

This checklist was used to assess students’ clinical skills in intraocular pressure (IOP) measurement using a non‑contact tonometer. This procedure was selected because it directly relates to the pathophysiology of glaucoma and the aqueous humor dynamics taught during the course. Students were required to perform the procedure on a simulated patient following standard clinical protocols. Two trained assessors, blinded to group allocation, scored each item using the criteria below. The total score was 100 points.

| Clinical Skills Assessment: Intraocular Pressure Measurement (Non‑Contact Tonometry) | | | |
| --- | --- | --- | --- |
| Item | Description and Scoring Criteria | Max Score | Score |
| Preparation |  | 10 |  |
| 1 | Confirm patient identity, introduce the procedure, obtain consent, and enquire about relevant history (e.g., corneal surgery, contact lens use, eye drops) | 3 |  |
| 2 | Explain the procedure to the patient and instruct the patient to sit comfortably with chin and forehead positioned against the instrument supports | 3 |  |
| 3 | Ensure the instrument is calibrated and the disposable probe cover is correctly installed | 4 |  |
| Examination Procedure |  | 75 |  |
| 4 | Position the patient correctly: chin on the chin rest, forehead against the forehead support, and eyes fixated on the internal target | 5 |  |
| 5 | Instruct the patient to open both eyes wide and blink normally; avoid holding breath or straining | 5 |  |
| 6 | Align the tonometer with the patient‘s eye using the joystick; centre the alignment target on the cornea | 10 |  |
| 7 | Instruct the patient to fixate on the internal light source and avoid blinking during measurement | 5 |  |
| 8 | Initiate the measurement and record the IOP reading displayed on the screen | 10 |  |
| 9 | Repeat the measurement three times and record the average value | 10 |  |
| 10 | Examine the fellow eye using the same procedure | 10 |  |
| 11 | Record the final IOP values (mmHg) for both eyes and note any abnormal readings | 10 |  |
| 12 | Identify clinically significant IOP elevation (e.g., > 21 mmHg) and relate it to the aqueous humor outflow pathway | 10 |  |
| Patient Care and Documentation |  | 15 |  |
| 13 | Provide appropriate aftercare instructions and reassure the patient | 5 |  |
| 14 | Record the procedure accurately in the patient record, including time, IOP values, and any complications | 5 |  |
| 15 | Demonstrate understanding of the normal IOP range and the clinical significance of elevated IOP in relation to glaucoma | 5 |  |
| Total |  | 100 |  |
| Assessor Signature: |  |  |  |

Critical Steps (▲)

▲ Patient identification – Failure to confirm patient identity before starting the procedure

▲ Correct eye identification – Examining the wrong eye or failing to confirm the eye to be tested

▲ Corneal alignment – Failure to correctly align the tonometer with the central cornea before measurement

▲ Proper positioning – Failure to position the patient correctly (chin and forehead against supports) before measurement

▲ Equipment calibration – Failure to confirm the tonometer is calibrated and ready for use

▲ Hand hygiene – Failure to perform hand hygiene before patient contact

▲ Chin rest hygiene – Failure to replace/disinfect the chin rest paper for each patient
